# Increased Serum Levels of S100A4 and S100A15 in Individuals Suffering from Hidradenitis Suppurativa

**DOI:** 10.3390/jcm10225320

**Published:** 2021-11-15

**Authors:** Aleksandra Batycka-Baran, Łukasz Matusiak, Danuta Nowicka-Suszko, Jacek C. Szepietowski, Wojciech Baran

**Affiliations:** Department of Dermatology, Venereology and Allergology, Wroclaw Medical University, 50-368 Wroclaw, Poland; lukasz.matusiak@umed.wroc.pl (Ł.M.); danuta.nowicka-suszko@umed.wroc.pl (D.N.-S.); wojciech.baran@umed.wroc.pl (W.B.)

**Keywords:** S100A4, S100A15, hidradenitis suppurativa

## Abstract

Hidradenitis suppurativa (HS) is a chronic inflammatory skin disease. Recently, some S100 proteins have been suggested to play an important role in the pathogenesis of chronic immune-mediated inflammatory diseases and they may constitute valuable biomarkers for these diseases’ diagnosis and monitoring. The objective of the current study was to investigate, for the first time, serum levels of S100A4 and S100A15 in individuals suffering from HS. Furthermore, we assessed the associations between S100A4 and S100A15 serum levels and the severity of disease, CRP serum concentration and some demographic and clinical data. Serum levels of S100A4 and S100A15 were evaluated with the commercially available ELISA kit according to the manufacturer’s instructions. The serum level of S100A4 in individuals with HS was significantly elevated as compared to controls, with the highest level found in the individuals in Hurley stage II. The S100A15 serum level was positively correlated with the CRP concentration and was associated with the severity of the disease. The serum level of S100A15 in the individuals in Hurley stage III was significantly elevated compared to that of the controls and the individuals with HS in Hurley stages I and II. S100A4 and S100A15 may be considered as new serum biomarkers for the monitoring of HS progression, and they may play a role in the pathogenesis of HS by promoting inflammatory process and fibrosis.

## 1. Introduction

Hidradenitis suppurativa (HS)/acne inversa is a chronic inflammatory and suppurative skin disease that affects intertriginous areas of the body, such as the axillary, inguinal and anogenital regions. It clinically manifests as tender nodules and abscesses; additionally, fistulas, fibrosis, scarring and dermal contractures may appear as the disease progresses [1,2,3]. The pathogenesis of HS is still not completely elucidated and requires further investigation. Recently, the important role of aberrant innate immune responses in epidermal abnormalities has been highlighted [3,4,5]. Some recent studies suggest that a defect in antimicrobial response may be present in individuals with HS, that may result from a deficiency of interleukin (IL)-20, IL-22 or the ineffective antimicrobial activity of IL-26 [6,7]. Further, HS is currently considered to be a chronic systemic inflammatory disease that is associated with metabolic and cardiovascular comorbidities [1,8,9,10]. The dignosis of HS is still usually made clinically. There are several scoring systems for the assessment of the disease’s severity, mainly based on clinical criteria. However, all of them have some limitations in daily practice. The Hurley classification system was first suggested in 1989, and it is still widely used [1,2]. In HS, the course and progression of the disease are difficult to predict. Currently, there is a lack of suitable plasma or serum biomarkers that could facilitate differentiation between stages and inform prediction of the progression of the disease [1,11]. S100 proteins belong to a family of low-molecular-weight (9–13 kDa), calcium-binding proteins that are involved in extra- and intra-cellular signaling and the regulation of cell proliferation and differentiation. Recently, some S100 proteins have been suggested to play an important role in the pathogenesis of chronic immune-mediated inflammatory diseases and, therefore, it is thought that they may constitute valuable biomarkers for use in diagnosis and monitoring [11,12,13]. Similarly, calprotectin (S100A8/S100A9) has been suggested to be a predictive biomarker of adalimumab response in HS patients [14]. As members of the S100 protein family, S100A4 and S100A15 are encoded within a frequently rearranged gene cluster, namely the epidermal differentiation complex on chromosome 1q21 [13,15,16]. S100A4 (metastasin, a fibroblast-specific protein) is well known to be involved in cancer progression and metastasis. However, S100A4 is also expressed in non-tumor cells (e.g., fibroblasts, activated lymphocytes, neutrophils and macrophages) and is involved in various non-malignant pathophysiologies, such as immune response, inflammation and angiogenesis. Furthermore, it promotes tissue fibrosis and is considered to be a specific fibroblast marker. S100A4 is involved in the pathogenesis of kidney and pulmonary fibrosis, as well as psoriasis, systemic sclerosis and hypertrophic scarring [16,17,18,19,20,21]. S100A15 was first identified from its overexpression in ‘koebnerized’ psoriatic skin; therefore, it was named koebnerisin [15]. Recently, an overexpression of S100A15 was found in the lesional and perilesional skin of individuals with HS compared to the skin of healthy controls [22]. S100A15 is an antimicrobial protein that reduces the survival rates of *Escherichia coli*, *Staphylococcus aureus* and *Pseudomonas aeruginosa*. It regulates keratinocyte proliferation and differentiation. Further, S100A15 has been identified as an endogenous danger-associated molecular pattern (DAMPS) with proinflammatory properties. S100A15 has been shown to prime keratinocytes for the enhanced production of proinflammatory cytokines, including TNF-α, IL-6 and IL-8, and it has been found to act as a chemotactic factor for neutrophils and monocytes/macrophages [15,23,24]. The objective of the current study was to investigate, for the first time, serum levels of S100A4 and S100A15 in individuals suffering from HS. Furthermore, we assessed the associations of S100A4 and S100A15 serum levels with the severity of HS, CRP serum concentration and some demographic and clinical data.

## 2. Materials and Methods

The study group consisted of 61 individuals suffering from HS (31 females/30 males) and 30 healthy controls (16 females/14 males) matched by age and sex. The diagnoses of HS were based on well-established clinical criteria (3). All patients with HS had active disease with the presence of inflammatory lesions (abscesses and nodules). HS patients were biologically naive and had not received any local or systemic anti-inflammatory therapy (e.g., antibiotics or retinoids) for at least 8 weeks prior to the initiation of the study. The age of subjects ranged from 19 to 67 years (mean ± SD = 38.5 ± 10.9). The mean duration of the disease was 8.7 ± 7.4 years, ranging from 2 to 27 years. The level of severity of disease was assessed according to the Hurley staging system; 19 individuals scored at stage I (31.1% of the HS cohort), 28 individuals scored at stage II (45.9%) and 14 individuals scored at stage III(22.9%). The mean BMI score for the HS cohort was 28.9 ± 4.9, with an obesity percentage of 42.6% (where obesity is defined as BMI ≥ 30, as per the WHO). The percentage of smokers among the HS cohort was 63.9%. The control group consisted of healthy subjects. The mean age was 39.3 ± 10.3 years and the mean BMI was 25.6 ± 5.8, with an obesity percentage of 20%. The percentage of smokers among the control cohort was 16.6%. Exclusion criteria included other skin diseases, chronic infection, inflammatory bowel disease, chronic kidney, liver or heart diseases and malignancies (Table 1).

From each subject, 5 mL of peripheral blood was taken. The assessment of serum concentrations of S100A4 and S100A15 was performed using a commercially available ELISA kit, according to the manufacturer’s instructions (MyBiosource Inc., San Diego, CA, USA).

The assessment of serum C-reactive protein (CRP) was performed using a turbidimetric assay on the Architect ci4100 analyzer (Abbott Diagnostics, Lake Forest, IL, USA). Informed consent was obtained from all participants. The study was conducted according to the Declaration of Helsinki and approved by the local Bioethical Committee.

### Statistical Analysis

The statistical analyses were performed using Statistica (version 13.1, StatSoft, Tulsa, OK, USA) and GraphPad Prism version 5.0. (La Jolla, CA, USA). The mean and standard deviations were calculated. Shapiro-Wilk and Kolmogorov–Smirnov normality tests were used to analyze whether variables were of normal or abnormal distribution. The significance of the differences between variables was determined using the Mann–Whitney U test, Pearson’s χ^2^ test, chi-square with Yates’ correction test and Fisher’s exact test and the Kruskal–Wallis test. Relationships between continuous variables of interest were assessed using Spearman’s rank correlation coefficient, and receiver operating characteristics (ROC) curve analysis was performed. Based on the ROC curve analysis, the area under the curve (AUC) and the optimal cut-off value were obtained. The optimal cut-off value was obtained from the Youden index (maximum (sensitivity + specificity − 1)), i.e., as a point with the highest sensitivity and specificity. A *p*-value < 0.05 was considered to be statistically significant.

## 3. Results

**Figure 1:**
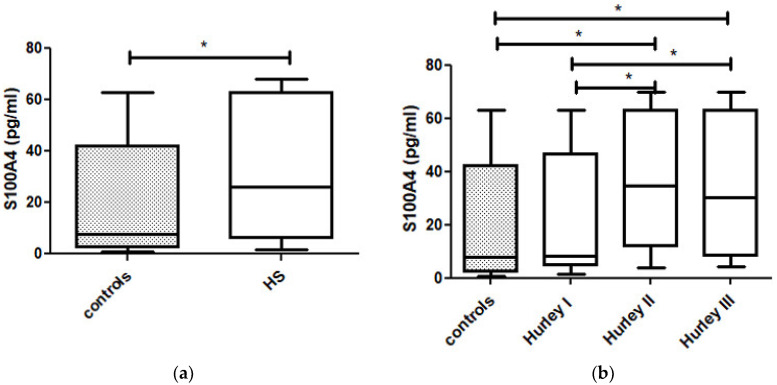
(**a**). The serum concentration of S100A4 in individuals suffering from HS was significantly elevated as compared to controls (*p* = 0.022). (**b**). There was statistically significant difference in S100A4 serum concentrations between Hurley stages (*p* = 0.03), with the highest concentration of S1004 in patients in Hurley II stage. The serum concentrations of S100A4 in patients in Hurley II stage was significantly elevated as compared to controls and individuals with HS in Hurley I stage (*p* = 0.005, *p* = 0.012, respectively). The serum concentrations of S100A4 in patients in Hurley III stage was significantly elevated as compared to controls and individuals with HS in Hurley I stage (*p* = 0.039, *p* = 0.049, respectively). * is statistically significant result *p* < 0.05.

The serum concentration of S100A4 in the individuals suffering from HS was significantly elevated when compared to those in the controls (31.57 pg/mL ± 24.38 vs. 22.51 pg/mL ± 23.30, *p* = 0.022) (Figure 1a). 

There was a statistically significant difference in the S100A4 serum concentrations between the three Hurley stages (*p* = 0.03), with the highest concentration of S1004 being found in patients in Hurley stage II (Figure 1b, Table 2). 

The serum concentration of S100A4 in the patients in Hurley stage II was significantly elevated compared to that of the controls and the individuals with HS in Hurley stage I (*p* = 0.005 and *p* = 0.012, respectively) (Figure 1b). Similarly, the serum concentrations of S100A4 in the patients with HS in Hurley stage III were significantly elevated as compared to those of the controls and the individuals with HS in Hurley stage I (*p* = 0.039 and *p* = 0.049, respectively) (Figure 1b). There was no significant difference in the serum concentrations of S100A4 between the individuals with HS in Hurley stages II and III (Figure 1b). We did not find any significant correlations between the serum concentrations of S100A4 and CRP, BMI, smoking habit or other demographic and clinical data.

**Figure 2:**
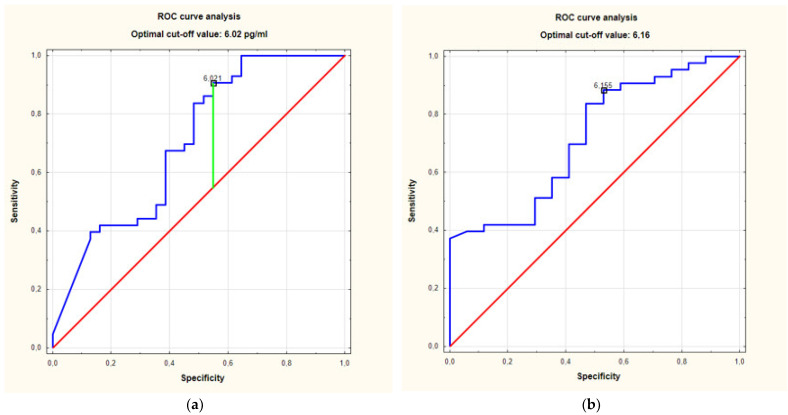
(**a**). ROC curve analysis of relationship between serum concentrations of S100A4 in individuals with HS in stage Hurley II + III and controls. Receiver operating characteristics (ROC) curves analysis showed area under the curve (AUC) of 0.7. (95%CI for AUC: 0.58–0.83) (*p* = 0.0014). (**b**). ROC curve analysis of relationship between serum concentrations of S100A4 in individuals with HS Hurley II + III and Hurley I. Receiver operating characteristics (ROC) curves analysis showed area under the curve (AUC) of 0.72 (95%CI for AUC: 0.58–0.86) (*p* = 0.0022).

The ROC analysis of the S100A4 serum concentrations in the individuals with HS in Hurley stage II and Hurley stage III, as compared to the controls, showed an area under the curve (AUC) of 0.7 (95%CI for AUC: 0.58–0.83) (*p* = 0.0014). The optimal cut-off value was set at 6.02 pg/mL (Figure 2a).

The ROC analysis of the serum concentrations of S100A4 in the individuals with HS in Hurley stage II and Hurley stage III, as compared to the individuals with HS in Hurley stage I, showed an AUC of 0.72. (95%CI for AUC: 0.58–0.86) (*p* = 0.0022). The optimal cut-off value was set at 6.16 pg/mL (Figure 2b). 

**Figure 3:**
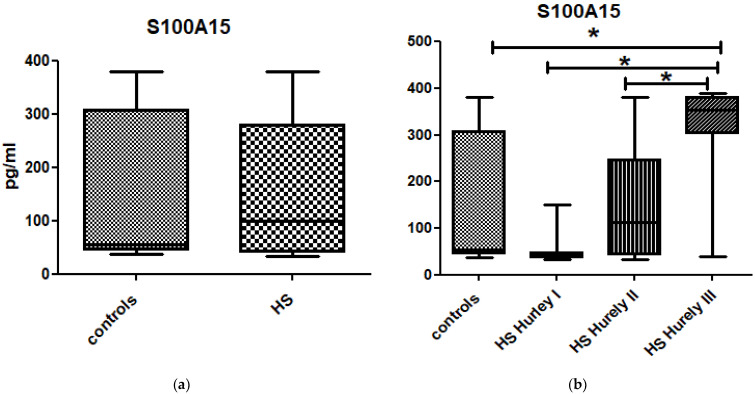
(**a**). There was no significant difference in serum concentrations of S100A15 between patients suffering from HS and controls (*p* > 0.05). (**b**). There was statistically significant difference in S100A15 serum concentrations between Hurley stages (*p* < 0.0001). The serum concentrations of S100A15 in patients in Hurley III stage were significantly elevated as compared to controls, individuals with HS in Hurley I stage and individuals with HS in Hurley II stage (*p* = 0.0013, *p* < 0.0001 and *p* = 0.0004 respectively). * is statistically significant result *p* < 0.05.

There was no significant difference in the serum concentration of S100A15 between the whole group of patients suffering from HS (156.1 ± 133.8 pg/mL) and the control group (153.9 ± 134.8 pg/mL) (*p* > 0.05) (Figure 3a). The S100A15 serum concentration was associated with the severity of disease as classified according to the Hurley staging system; there was a statistically significant difference in S100A15 serum concentrations between the Hurley stages (*p* < 0.0001), with the highest concentration of S10015 being found in the patients in Hurley stage III (Figure 3b, Table 2). The serum concentration of S100A15 in the patients in Hurley stage III was significantly elevated compared to those in the controls (*p* = 0.0013) (Figure 3b).

**Figure 4:**
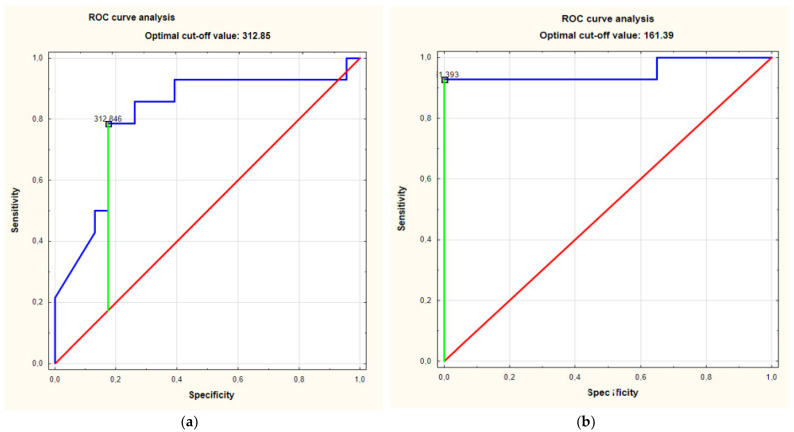

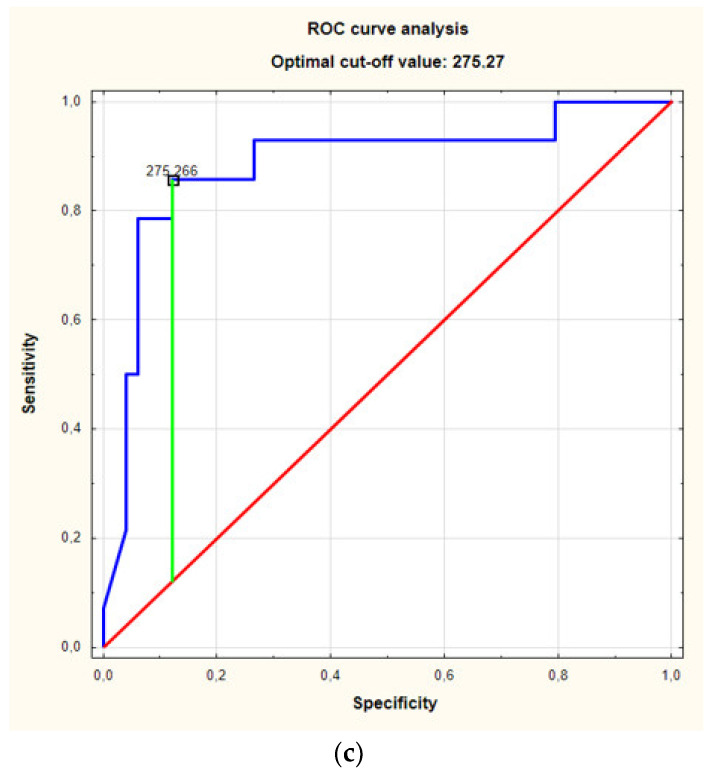
(**a**). ROC curve analsysis of relationship between serum concentrations of S100A15 in individuals with HS in stage Hurley III and controls showed area under the curve (AUC) of 0.81. (95%CI for AUC: 0.66–0.97) (*p* = 0.0001). (**b**). ROC curves analysis of relationship between serum concentrations of S100A15 in individuals with HS in Hurley III stage and in individuals with HS in Hurley I stage revealed AUC of 0.95 (95%CI for AUC: 0.86–1) (*p* < 0.0001). (**c**). ROC curves analysis of relationship between serum concentrations of S100A15 in individuals with HS in Hurley III stage and in individuals with HS in less severe stages (Hurely I, Hurley II) revealed AUC of 0.88 (95%CI for AUC: 0.77–0.99) (*p* < 0.0001).

The ROC curve analysis showed an AUC of 0.81 (95%CI for AUC: 0.66–0.97) (*p* = 0.0001). The optimal cut-off value was set at 312.85 pg/mL (Figure 4a).

The serum concentrations of S100A15 in the individuals with HS in Hurley stage III were significantly increased compared to those of the individuals with HS in Hurley stage I and the individuals with HS in Hurley stage II (*p* < 0.0001 and *p* = 0.0004, respectively) (Figure 3b). The ROC curve analysis of the relationship between the serum concentration of S100A15 in the individuals with HS in Hurley stage III and in the individuals with HS in Hurley stage I revealed an AUC of 0.95 (95%CI for AUC: 0.86–1) (*p* < 0.0001). The optimal cut-off value was 161.39 pg/mL (Figure 4b).

The ROC curve analysis of the relationship between the serum concentration of S100A15 in the individuals with HS in Hurley stage III and in the individuals with HS in the less severe stages (Hurley I and Hurley II) revealed an AUC of 0.88 (95%CI for AUC: 0.77–0.99) (*p* < 0.0001). The optimal cut-off value was 275.27 pg/mL.

**Figure 5:**
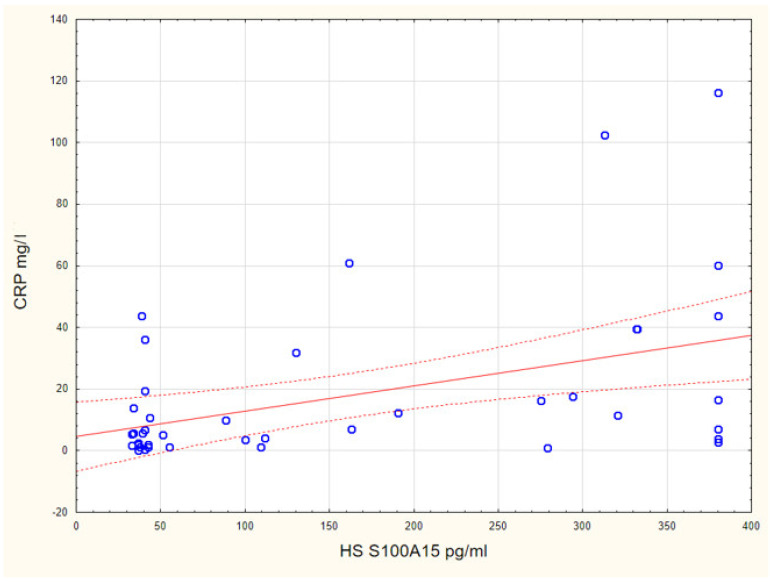
The serum concentration of S100A15 in individuals with HS was positively correlated with CRP concertation (*p* = 0.004, *R* = 0.425).

The serum concentration of S100A15 in the individuals with HS was positively correlated with their CRP concentration (*p* = 0.004, *R* = 0.425) (Figure 5). We did not find any correlations between the serum concentrations of S100A15 and BMI, smoking habit or other demographic and clinical data.

## 4. Discussion

To the best of our knowledge, this is the first study to evaluate the serum levels of S100A4 and S100A15 in individuals with HS. We found significantly elevated serum S100A4 levels in the individuals with HS as compared to the controls. The highest serum level of S1004 was found in the patients with HS in Hurley stage II. S100A4 exerts proinflammatory action and has been shown to be involved in the pathogenesis of some chronic immune-mediated inflammatory skin diseases, such as psoriasis and systemic sclerosis [16,17,18,19,20]. A weak and sparse expression of S100A4 was shown in the normal skin tested. Upon various proinflammatory stimuli, numerous inflammatory cells (e.g., lymphocytes, neutrophils and macrophages), fibroblasts and endothelial cells up-regulate the expression of S100A4 by releasing it into the extracellular space. S100A4 is an active extracellular factor with the capacity to influence gene expression by modulating numerous signaling pathways and transcription factors, including NF-κB. S100A4 also induces the expression of proinflammatory cytokines, such as IL-1β, IL-6 and TNF-α, acute phase reactants and some other S100 protein family members (e.g., 100A8 and S100A9) which are known to be involved in the pathogenesis of HS [1,11,16,25,26]. Further, it promotes the recruitment and chemotaxis of inflammatory cells [16,17,19,20,21]. As a result of these known processes, S100A4 may enhance inflammatory process in HS. In the present study, we did not find any correlation between S100A4 serum level and CRP serum level. HS is associated with tissue remodeling [1], while S100A4 is considered to be a fibroblast marker, used to predict and monitor fibrosis in various tissue. In response to persistent inflammation, S100A4 induces tissue fibrosis and is involved in the transition of epithelial or endothelial cells into inflammation-induced fibroblasts. Further, it stimulates fibroblasts for the aberrant production of extracellular matrix (ECM) and induces the expression and activation of matrix metalloproteinases (MMPs) [16,17,18]. The course and progression of HS is hard to predict. All patients start in Hurley stage I and some of them progress to more advanced stages in which sinus tracts, fibrosis, scarring and dermal contractures occur [1,2]. Therefore, there is a need for the identification of biomarkers that may be useful in monitoring the progression of HS [1,11]. In the present study, the serum S100A4 levels in the individuals with HS in Hurley stages II and III were significantly increased compared to those in the individuals in Hurley stage I. The ROC curve analysis showed the AUC to be 0.72. The optimal cut-off point was set at 6.16 pg/mL. Therefore, we suggest that the serum level of S100A4 may serve as a biomarker to predict and monitor inflammatory processes and fibrosis in individuals with HS; in particular, S100A4 may facilitate differentiation between stage I and the more advanced stages according to Hurley classification. Further research is required to establish the role of S100A4 in the pathogenesis of HS and its usefulness as a serum marker in daily practice.

In the present study, we found elevated serum levels of S100A15 in the individuals with HS in Hurley stage III compared to those in the controls. The S100A15 serum levels in the individuals with HS were positively correlated with their CRP serum levels. S100A15 has a proinflammatory function and is known to potentiate inflammatory processes in the skin [15,22,23,24]. Furthermore, it has been shown to stimulate mononuclear cells circulating in peripheral blood, including lymphocytes and monocytes, for the increased expression and production of proinflammatory cytokines, such as TNF-α, IL-1β, IL-6 and IL-8 [27]. It also acts as a chemotactic factor for neutrophils and monocytes/macrophages [24]. TNF-α, Il-1β, IL-6 and IL-8 are involved both in the pathogenesis of HS as well as in the development of glucose intolerance, obesity, hyperlipidemia, metabolic syndrome and atherosclerosis [1,3,4,9]. Up-regulated by proinflammatory micromilieu, S100A15 may amplify both skin and systemic inflammation and contribute to the development of comorbidities in individuals with HS. Increased serum levels of S100A15 have also been found in patients suffering from psoriasis, and it has been recently proposed as a biomarker of subclinical atherosclerosis [28]. Recent studies have indicated that HS is linked with an increased risk of major adverse cardiac events (MACEs), which is even higher than the risk of MACE that is associated with psoriasis. Further, an increased frequency of subclinical atherosclerosis has been found in patients with HS [9,29]. In the present study, we found a positive correlation between the S100A15 serum level and the CRP level in the individuals with HS. It is suggested that the link between HS and increased cardiovascular risk results from chronic systemic inflammation [9,29]. The elevated level of CRP is considered to be a marker of inflammatory processes and an independent cardiovascular risk factor [30]. We did not find a correlation between S100A15 level and BMI or smoking habit. The observed serum level of S100A15 was significantly elevated in the individuals with HS in Hurley stage III compared to that in the individuals with HS in Hurley stage I. The ROC curve analysis revealed a large AUC of 0.95. The optimal cut-off value was set at 161.39 pg/mL. Therefore, the serum level of S100A15 may be suggested as a marker for the monitoring of the progression of HS. A limitation of this study is the small cohort of patients. The independent influences of smoking and BMI on the obtained results were not estimated. Further research is required to establish the relevance of S100A15 as a serum marker of systemic inflammation and subclinical atherosclerosis in individuals with HS.

Here, we suggest new serum biomarkers for the monitoring of HS progression. S100A4 and S100A15 may be involved in the pathogenesis of HS by promoting inflammatory processes and fibrosis.

## Figures and Tables

**Table 1:** Characteristics of population.

		Patients			Controls	*p* Value
Gender F/M		31/30			16/14	*p* > 0.05
Age (years)		38.5 ± 10.9			39.3 ± 10.3	*p* > 0.05
BMI (kg/m^2^)		28.9 ± 4.9			25.6 ± 4.2	* *p* < 0.05
Disease duration (years)		8.7 ± 7.4			N/A	
CRP (mg/L)		17.4 ± 25.2			2.7 ± 2.0	* *p* < 0.05
Smokers/non-smokers		39/22			5/25	* *p* < 0.05
Hurley staging		Females	Males	Total	N/A	
	Hurley I	12	7	19		
	Hurley II	15	13	28		
	Hurley III	4	10	14		

* *p* < 0.05 statistically significant.

**Table 2:** Serum levels of S100A4, S100A15 and C-reactive protein (CRP) in healthy controls and patients with hidradenitis suppurativa, according to Hurley staging system.

	N	S100A4 (pg/mL) Mean ± SD	S100A15 (pg/mL) Mean ± SD	CRP (mg/L) Mean ± SD
Controls	30	22.5 ± 23.3	153.9 ± 134.0	2.7 ± 2.0
Hurley I	19	22.7 ± 22.6	50.8 ± 30.9	3.9 ± 4.4
Hurley II	28	37.6 ± 24.5	151.5 ± 115.7	9.6 ± 10.1
Hurley III	14	35.3 ± 24.9	317.1 ± 101.0	43.1 ± 35.2

## Data Availability

All source data are available per request by email.
